# COVID-19 Virus Infection in Three Patients With Hypogammaglobulinemia

**DOI:** 10.7759/cureus.15256

**Published:** 2021-05-26

**Authors:** Quinto Gesiotto, Asima Cheema, Kishan Avaiya, Bijal Shah, John Greene

**Affiliations:** 1 Internal Medicine, University of South Florida Morsani College of Medicine, Tampa, USA; 2 Infectious Disease, University of South Florida, Tampa, USA; 3 Medical Education, University of Florida, Gainesville, USA; 4 Malignant Hematology, Moffitt Cancer Center, Tampa, USA; 5 Internal Medicine, Moffitt Cancer Center, Tampa, USA

**Keywords:** covid 19, hypogammaglobulinemia, multiple myeloma, lymphoma, mantle cell lymphoma, intravenous immunoglobulin (ivig)

## Abstract

The world is experiencing the COVID-19 outbreak and there are no evidence-based treatment strategies available for immunocompromised patients. COVID-19 is a novel beta coronavirus known as severe acute respiratory syndrome coronavirus 2 (SARS-CoV-2). Patients with cancer are more susceptible to infection than individuals without cancer due to their impaired humoral and cellular immune function caused by the malignancy itself and chemotherapy. We present three cases of cancer patients with hypogammaglobulinemia with varying clinical outcomes associated with infection. These include one mantle cell lymphoma patient with recurrent respiratory infection requiring intravenous immunoglobulin (IVIG) support and two multiple myeloma patients with continued viral shedding.

## Introduction

The COVID-19 pandemic has caused severe morbidity and mortality and an unprecedented burden on health care systems globally [1]. Patients with hypogammaglobulinemia and/or underlying malignancy, such as non-Hodgkin lymphoma (NHL) or multiple myeloma, may be at particularly high risk of infection and poor outcomes related to COVID-19. A detailed analysis of outcomes for these patients, particularly examining the effects of baseline characteristics and lymphoma or multiple myeloma-directed therapies, is critical to optimally manage this population through this evolving pandemic [2].

## Case presentation

Patient 1

The patient is a 63-year-old male diagnosed with stage IVB mantle cell non-Hodgkin's lymphoma (MCL) in April 2011. He was treated with five cycles of HyperCVAD (cyclophosphamide, vincristine, adriamycin, and dexamethasone), followed by autologous stem cell transplantation in October 2011 with continuous complete remission. The post-transplant course was notable for hypogammaglobulinemia requiring IVIG replacement. In May 2020, he presented to hematology clinic and reported multiple recent upper respiratory infections in the preceding six months requiring antibiotics. Of note, his last IVIG infusion had been a few days prior and his immunoglobulin G (IgG) levels were 379 in November and less than 300 in May 2020. On physical examination, vitals were normal, oropharynx clear, normal hearing, oral mucosa moist, neck supple, and no lymphadenopathy. Heart rate normal and regular rhythm, lungs clear to auscultation bilateral, abdomen soft, non tender, no organomegaly, no lower extremity edema, skin warm and dry. Laboratory data revealed white blood cell (WBC) at 7.32, absolute neutrophil count (ANC) > 1.5 k/µL, Hb at 14.9, platelet count 171 k/µL, LDH was 183 U/L, immunoglobulins were < 300 mg/dL (low), immunoglobulin A (IgA) 122 mg/dL, and immunoglobulin M (IgM) 59 mg/dL. PET/CT scan of the chest revealed calcified hilar and mediastinal lymph nodes and calcified lung nodules with a small amount of peripheral ground glass infiltrate along the lateral left lower lobe (image 95) with standardized uptake value (SUV) maximum 2.7 compared to 2.0 previously. He complained of fever, cough, nasal congestion and anosmia but no shortness of breath. COVID-19 nasopharyngeal polymerase chain reaction (PCR) testing was positive on August 28, 2020. 

The patient continued to experience cough, weakness, and fatigue for another week; however, his anosmia and subjective fevers subsided. He presented to hematology clinic on September 4, 2020 and received a 25 g IVIG infusion. On September 10, 2020 he had a repeat COVID-19 nasopharyngeal swab test which was inconclusive, and he presented on October 3, 2020 for a second IVIG infusion. His symptoms continued to improve and he underwent staging PET/CT in November 2020 showing interval decreases in the neck and tonsillar lymph node metabolic activity and persistent metabolic activity in paratracheal, precarinal, and hilar lymph nodes consistent with stable disease. At his follow-up appointment in January 2021, he was feeling well with only a complaint of night sweats, and resolution of his weakness, fatigue, anosmia, and fevers. Vital signs were negative for fever and his oxygen saturation was 97% on room air. He was started on another six-month course of monthly IVIG and has continued recovery from COVID-19. He received his first dose of the Pfizer COVID-19 vaccine on March 27, 2021.

Patient 2

The patient is a 49-year-old female diagnosed with IgG kappa multiple myeloma in May 2019. She was treated initially with Velcade and dexamethasone for four cycles (Revlimid was held due to lactose intolerance) and then in September 2019 was transitioned to Kyprolis, Revlimid, and dexamethasone for six cycles; however, she was found to have progressive disease. She was then bridged to carfilzomib combined with cyclophosphamide and dexamethasone; however, after only one cycle she presented to the hospital on April 6, 2020 with fevers and cough for two days. On physical exam, she was febrile but maintaining normal oxygen saturation. Labs were significant for a WBC count of 3.5x10^3^ per millimeter cube, C-reactive protein 9.1 mg/dL, and ferritin 94.6 nanograms per milliliter (ng/mL). Nasopharyngeal PCR swab for COVID-19 was positive. Chest X-ray and CT were unremarkable at the time with no signs of pneumonia. The patient was admitted for two days and discharged on a course of oral azithromycin. She reported back for a repeat COVID-19 test on April 20, 2020 and was asymptomatic at the time; however, the repeat test was positive.

The patient continued to have no symptoms and remained quarantined at home. Myeloma labs remained stable at subsequent hematology appointments. Plans were made to start chimeric antigen receptor T-cell (CAR-T) therapy; however, per CDC guidelines she required negative COVID-19 testing prior to initiation. She presented for repeat COVID-19 testing on April 27, 2020, May 17, 2020, and May 26, 2020, all of which were positive. Testing on June 5, 2020 and July 6, 2020 was negative. During this time, the patient presented to hematology clinic and was found to have progression of her myeloma with rise in kappa light chains, hypercalcemia, and worsening renal insufficiency. She was given Zometa and the pain control regimen was increased. Restaging bone marrow biopsy on July 9, 2020 showed 70% plasma cells. She continued to have no COVID-19-related symptoms but unfortunately tested positive on July 9, 2020. She was not initiated on COVID-19 therapy (such as monoclonal antibodies or steroids) as she was determined to be an asymptomatic shedder.

Due to continued positive testing preventing CAR-T enrollment and progressive disease, she was started on chemotherapy with carfilzomib, cyclophosphamide, and dexamethasone on July 10, 2020. Restaging labs after chemotherapy showed a decline in kappa light chains. The patient then tested negative for COVID-19 on July 31, 2020, August 4, 2020, and August 11, 2020 and began autologous anti-B-cell maturation antigen (anti-BCMA) CAR-T therapy on August 18, 2020 with plans for a repeat bone marrow biopsy 90 days after initiation. As of March 2021, she remains in stringent complete response and has received both doses of the COVID-19 vaccine without complication or reinfection.

Patient 3

The patient is a 71-year-old male diagnosed with IgG lambda multiple myeloma in June 2011 after he was noted to have elevated total protein levels on routine labs. He was initially started on bortezomib, Revlimid, and dexamethasone in August 2012 and completed eight cycles, then was transitioned to Revlimid maintenance therapy. He underwent an autologous stem cell transplant on June 19, 2013 and resumed Revlimid maintenance therapy in October 2013; however, due to financial difficulties and inability to obtain the drug he continued on observation starting January 2017. Unfortunately, the patient’s disease progressed and he was started on daratumumab, Revlimid, and dexamethasone on June 23, 2017, although Revlimid was later discontinued in September 2017 due to cost. The patient again had progressive disease and bortezomib was added on December 15, 2017. The disease continued to slowly progress and therapy was transitioned on April 12, 2019 to carfilzomib with weekly dexamethasone for 11 cycles, then to maintenance carfilzomib on March 7, 2020.

On June 27, 2020, the patient began experiencing severe nausea, diarrhea, vomiting, fever, and malaise. He presented to the hospital on June 29, 2020 and was found to be febrile with a temperature of 100.6 F. He was saturating 98% on room air. Physical exam showed an ill-appearing male with clear lungs, normal heart rate, and no oropharyngeal erythema. Labs showed WBC 5.19x10^3^/mL^3^, lymphopenia with total lymphocytes 0.16x10^3^/mL^3^, platelet count of 55, creatinine of 1.2 mg/dL, high sensitivity CRP 5.3 mg/dL, ferritin 3,252 ng/mL, D-dimer 0.8 mg/dL, interleukin-6 3.6 pg/mL. COVID-19 nasopharyngeal swab was positive. Chest X-ray showed clear lungs bilaterally without any consolidations, effusions, or pneumothorax. He was admitted for five days, required no COVID-19-specific therapy and was discharged in stable condition with instructions to self-quarantine at home.

The patient again presented to the hospital on July 20, 2020 with right-sided pleuritic chest pain and shortness of breath for 10 days. On presentation, he was afebrile with oxygen saturation of 92% on room air. Physical exam showed decreased breath sounds and pain with deep inspirations. Labs were significant for D-dimer 52 mg/L, CT angiogram showed a saddle-type pulmonary embolism with involvement of the right greater than the left pulmonary arterial tree, no evidence of right heart strain. He was started on a heparin drip and admitted to the intensive care unit. Lower extremity Doppler ultrasounds showed deep vein thrombosis (DVT) in the R common femoral and femoral veins as well as the left femoral and popliteal veins. He was transferred out of the ICU on July 21, 2020 and was transitioned from heparin to Eliquis. Repeat COVID-19 testing was negative on August 8, 2020 by nasopharyngeal swab. He received his COVID-19 vaccine in February of 2021 and experienced migraines following the first dose but tolerated the second dose without any side effects. He resumed therapy with carfilzomib and of March 2021 his bone marrow continued to show a stable clonal population of plasma cells.

## Discussion

The natural history of COVID-19 infection in patients with hypogammaglobulinemia and/or malignancy is unknown, and therefore there are no available guidelines for the management of these patients who may be at increased risk of poor outcomes. Patients with hypogammaglobulinemia, especially in those with hematologic malignancies, may also experience delays in diagnosis and treatment of their primary disease due to continued viral shedding and complications related to their viral infection. Therefore, disease management in this population, particularly in the setting of concomitant chemotherapy or immunotherapy, requires further study.

The data suggest that among patients with cancer infected with COVID-19, 30-day all-cause mortality is high and associated with more severe outcomes when compared to patients without cancer [3]. Patients with hematological cancer, lung cancer, and cancers in metastatic stages demonstrated higher rates of severe events compared to patients without cancer [4,5]. In addition, patients who underwent cancer surgery showed higher death rates and higher chances of having critical symptoms. Longer follow-up generally is needed to better understand the effect of COVID-19 on outcomes in patients with cancer, including the ability to continue specific cancer treatments [3,5]. Patients with hematological malignancies, in particular, have worse outcomes than both the general population with COVID-19 and patients with hematological malignancies without COVID-19 [4]. Currently, around 15 million people are living with cancer [6]; however, little is known about the burden of COVID-19 in cancer patients who are at increased risk of worse outcomes [7]. The available clinical and biochemical data of COVID-19 might be partly masked by coexisting lymphoma; better diagnostic strategies such as superior CT differential techniques (i.e. high-resolution CT) could be used for diagnosis; individuals with compromised immune status might be subjected to a longer incubation period (although the underlying mechanisms are not known); and it remains uncertain whether the combination of chemotherapy, corticosteroids, α-interferon, and immunoglobulins could work synergistically in patients with NHL and COVID-19 [8]. Patients with cancer with different tumor types have differing susceptibility to SARS-CoV-2 infection and COVID-19 phenotypes, considering age, sex, and tumor subtype [6].

Patients with plasma cell disorders may experience more severe COVID-19 disease courses and unique complications; however, data about these patient populations are limited. A Spanish study of 216 hospitalized multiple myeloma patients with COVID-19 reported 50% higher mortality when compared to noncancer patients [9]. A study of 58 patients with smoldering myeloma and multiple myeloma with COVID-19 at Mount Sinai Hospital found that myeloma therapy and immunoparesis did not affect outcomes and that survival was comparable to the overall population of the area [10]. In a survey of 12 cancer centers in Belgium, 20 symptomatic multiple myeloma patients were identified, 16 of whom were on immunomodulatory therapy at the time, eight were post-transplant, and six with hypogammaglobulinemia [11]. In this cohort, hospitalization was required in 90% and severe or critical disease was reported in 75%, and mortality was 35%, indicating an aggressive clinical course in these patients. In a retrospective study of seven multiple myeloma patients with COVID-19 at Medical College of Wisconsin including two with hypogammaglobulinemia, the mortality rate was high with three patients dying during hospitalization and one dying within 10 days of discharge [12]. Both studies suggested that common presenting symptoms were fever and shortness of breath, consistent with the general population, but with much higher rates of hospitalization. COVID-19 was not shown to be associated with additional risks or poor outcomes in a seven-patient case series at Albert Einstein [13]. A five-patient study at MD Anderson reported five multiple myeloma patients with COVID-19, four with hypogammaglobulinemia, and reported no deaths; however, two patients experienced greater than two weeks of hospital stay and required mechanical ventilation [14]. A study of eight myeloma patients undergoing treatment in Sweden suggests that negative effects from COVID-19 in this population may be related to treatment-related immune dysregulation rather than disease-related immunoparesis or hypogammaglobulinemia [15]. Current data indicate that serologic assays for COVID-19 do not cross-react with the M-components seen in multiple myeloma [16].

There is no strong evidence to indicate that COVID-19 pathogenesis is significantly different in patients with hypogammaglobulinemia or multiple myeloma. There is one report of coinfection with COVID-19 and *Mycobacterium abscessus* in a patient with multiple myeloma who was eventually treated with convalescent plasma and remdesevir; however, there is no evidence to indicate that coinfection is more common in these patients [17]. While a recent report details a patient in Brazil with concern for COVID-19 reinfection, it is difficult to tell if cancer patients are at increased risk for reinfection and if reinfection can be confirmed with our testing [18].

The COVID-19 pandemic has also caused an unprecedented disruption throughout the cancer research community and has slowed down cancer clinical trial operations [19,20]. Once normal service resumes at a population and health-service level, there will be a huge backlog of patients with potential cancer symptoms needing urgent assessment [21]. There is limited data on immunosuppressed patients but early published reports from China on the outcomes of patients with cancer infected with COVID-19 indicated a 3.5 times higher risk of needing mechanical ventilation or ICU admission or dying compared with patients without cancer [22].

The development and widespread administration of novel vaccinations against COVID-19 have recently provided a means of protecting vulnerable patients, such as immunocompromised individuals and cancer patients. As of now, vaccines are recommended for all individuals with multiple myeloma without any other contraindications; however, there is very limited data on the available vaccines in these populations. One recent study of 67 patients with hematologic malignancies suggests that vaccine response in this population may be suboptimal, especially in B-cell chronic lymphocytic leukemia (CLL) patients, and that post-vaccination antibody measurements may be warranted [23]. We recommend that all patients with hypogammaglobulinemia (including multiple myeloma, CLL, and NHLs) should undergo vaccination for COVID-19; however, they may experience a blunted immune response. In these patients, testing for antibodies against the COVID-19 viral capsid may be warranted to determine if they have established protective immunity as some preliminary data suggest low neutralizing antibody response in elderly myeloma patients [24].

## Conclusions

In conclusion, patients with hypogammaglobulinemia secondary to multiple myeloma or lymphoma are at risk for severe infection with COVID-19. They are also more likely to shed the virus for a prolonged period which will delay needed therapy to treat the underlying hematologic malignancy, as evidenced in Patient 2 in our study who could not get CAR-T therapy due to continued positive COVID-19 testing and progressed on an alternative therapy. IVIG may be beneficial in hypogammaglobulinemia and indeed Patient 1 in our study experienced improvement of COVID-19 symptoms following IVIG infusion. Vaccination against COVID-19 is imperative in this population but the development of protective immunity and its duration are limited due to immunodeficiency, which yields hypogammaglobulinemia.
